# Exclusive enteral nutrition combined with continuous succus entericus reinfusion for high-output stoma in patients with Crohn’s disease: a case report

**DOI:** 10.1093/gastro/goae100

**Published:** 2024-10-27

**Authors:** Na Diao, Wenyou Zheng, Huiping Chen, Jian Tang

**Affiliations:** Department of Gastroenterology, The Sixth Affiliated Hospital, Sun Yat-sen University, Guangzhou, Guangdong, P. R. China; Biomedical Innovation Center, The Sixth Affiliated Hospital, Sun Yat-sen University, Guangzhou, Guangdong, P. R. China; Department of Gastroenterology, People’s hospital of Taishan, Jiangmen, Guangdong, P. R. China; Department of Gastroenterology, The Sixth Affiliated Hospital, Sun Yat-sen University, Guangzhou, Guangdong, P. R. China; Biomedical Innovation Center, The Sixth Affiliated Hospital, Sun Yat-sen University, Guangzhou, Guangdong, P. R. China; Department of Gastroenterology, The Sixth Affiliated Hospital, Sun Yat-sen University, Guangzhou, Guangdong, P. R. China; Biomedical Innovation Center, The Sixth Affiliated Hospital, Sun Yat-sen University, Guangzhou, Guangdong, P. R. China

## Introduction

Crohn’s disease (CD) is a chronic and recurrent inflammatory disease of the gastrointestinal tract. Even though the therapeutic landscape for CD has been revolutionized and surgical resection rates are generally on a downward trend over the past decades [1]. There are still up to 75% of patients with CD who will require intestinal resection at some point in their disease course, and the reported annual surgical rate ranges between 3.3% and 7.5% [2, 3]. Among patients undergoing resection surgery, some are prone to develop short bowel syndrome and high-output stoma (HOS), which were caused by surgery-induced reduction in bowel length, lacking ileocolonic continuity with an ostomy, and uncontrolled inflammation of the remaining small bowel. In this situation, patients may not tolerate a normal diet and may develop malnutrition, dehydration, electrolyte disturbances, and kidney failure due to HOS. Previous management of the malabsorption associated with HOS varies with the degree of severity, mainly including continuous parenteral nutrition, drug suppression of intestinal peristalsis, or anti-inflammatory treatment for the disease itself [4, 5]. Here, we shared a successful experience with exclusive enteral nutrition (EEN) and continuous succus entericus reinfusion (SER) to manage HOS in a patient with CD after extensive small bowel resection.

## Case presentation

**Figure 1.**
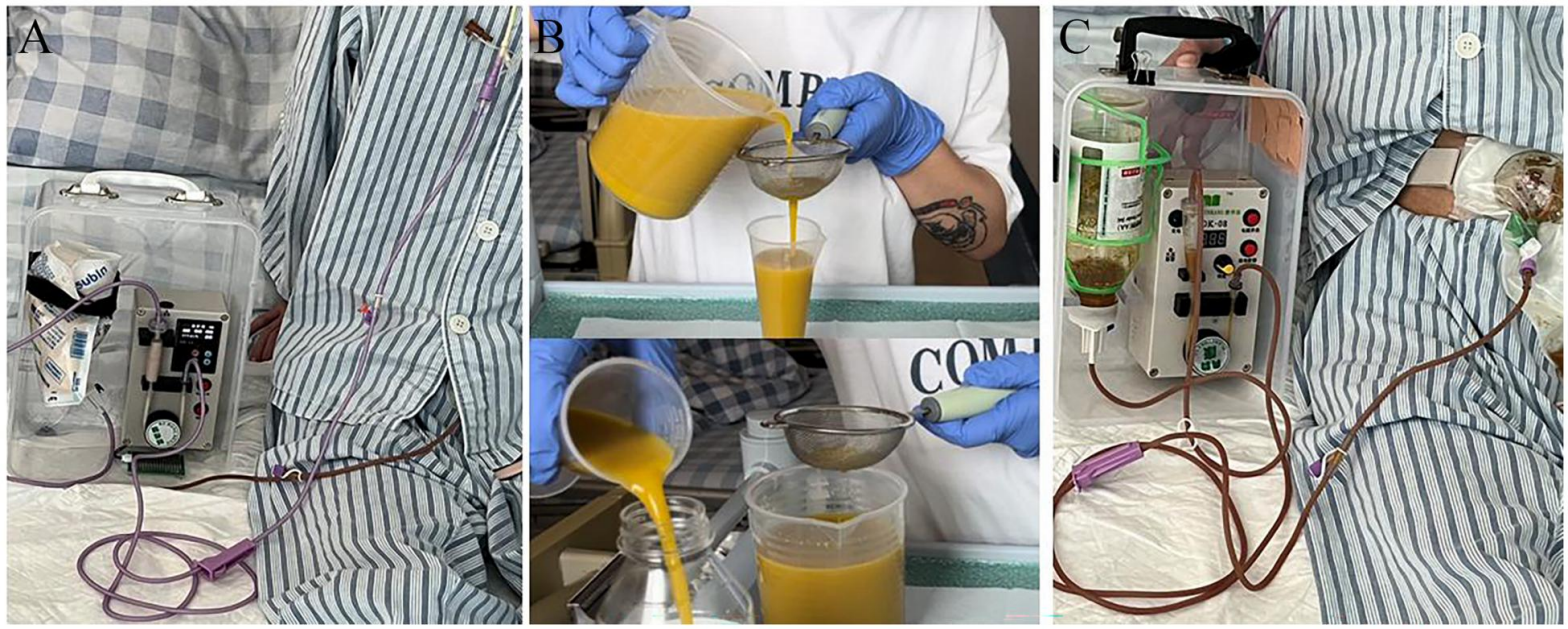
Clinical practice of exclusive enteral nutrition treatment and continuous succus entericus reinfusion. (**A**) Step 1: exclusive enteral nutrition treatment via nasogastric tube. (**B**) Step 2: collect jejunostomy effluent and filter out solid matter. (**C**) Step 3: pump jejunostomy effluent into the distal ileostomy.

A 35-year-old male patient with CD, complicated with chronic intestinal obstruction due to intestinal fistulas and multiple small intestinal stenoses, was admitted to our center. The patient underwent partial small intestine resection and double barrel ostomy of the jejunum and ileum, with 160 cm of residual jejunum above the ostomy and 100 cm of residual ileum below the ostomy. The patients resumed to normal diet after surgery and developed a HOS with daily jejunostomy output exceeding 2,000 mL, accompanied by fatigue and oliguria, requiring continuous intravenous nutrition therapy. To manage the HOS, the patient was given EEN therapy via nasogastric tube with daily energy intake of 1,500–2,000 kcal, fluid intake of 2,000 mL, and jejunostomy effluent of 2,000–2,500 mL. A catheter was placed ∼15 cm into the patient’s ileostomy, and an enteral feeding pump was used to continuously and slowly pump jejunostomy effluent (1,000–1,500 mL/day) into the distal ileostomy (Figure 1). The patient and his family member were instructed to collect the jejunostomy effluent, filter out solid matter, and manage reinfusion pump by themselves. One week after treatment with EEN and continuous SER, the patient was able to discontinue intravenous nutrition and was discharged, with bowel movements of 0–1 time per day, urine output stable at around 1,000 mL per day, and weight gain about 1 kg. After discharge, the patient persisted with treatment of EEN and SER at home and his BMI increased from 13.6 to 16.0 kg/m^2^ on 1 month and to 19.5 kg/m^2^ on 6 months.

## Follow-up

At 6 months after surgery, he returned to the hospital for re-examination. CTE displayed no obvious narrowing or obstruction and a markable improvement of inflammation of residual intestine (Supplementary Figure 1). Colonoscopy revealed the mucosal lesion was healing. Therefore, the stoma closure was successfully performed, and ustekinumab was used postoperatively to further control the intestinal inflammation.

## Discussion

Patients with CD sometimes are confronted with some unfortunate complications, such as short bowel syndrome and HOS, after major abdominal surgery requiring extensive small bowel resection and high position enterostomy [6]. SER is the standard treatment for general HOS patients [6], but traditional SER implemented in CD patients has certain difficulties, including: (i) CD patients may have lesions in the distal intestine, and SER after normal diet may reduce the therapeutic effect of original diversion; (ii) CD patients may have stenosis in the small intestine and colon distal to the stoma, and stoma output containing too much solid content, which may cause obstruction; (iii) CD patients may have lower distal intestinal absorption capacity than ordinary patients, and the use of syringes for pulse SER may lead to abdominal distention and diarrhea. Only by solving the above problems can SER be better used in CD patients.

EEN is an effective treatment for pediatric and refractory adult CD [7]. Continuous EEN administration via nasogastric tube not only supplements nutrients for patients with short bowel syndrome but also improves absorption and suppresses inflammation of residual intestinal caused by CD. Moreover, the jejunostomy effluent contains no solid matter and is easy to perform SER. Notably, the jejunostomy effluent after EEN reinfused into the distal residual ileum not only promotes the absorption of nutrients and water but also mimics the continuity of the digestive tract, helping to inhibit inflammation in the distal residual intestine and colon.

Here, we describe for the first time that EEN plus continuous SER can effectively treat HOS in patients with CD, while also addressing the intestinal inflammation of CD, reducing the usage of perioperative intravenous nutrition and biologics or immunosuppressive drugs. This technique has the potential to become a standardized treatment for CD patients with HOS during the perioperative period.

## Supplementary Material

goae100_Supplementary_Data
