# Supplementary material for: Exclusive enteral nutrition combined with continuous succus entericus reinfusion for high-output stoma in patients with Crohn’s disease: a case report
Source: Gastroenterol Rep (Oxf). 2024 Oct 27;12:goae100. doi: 10.1093/gastro/goae100 (PMC11513195; doi:10.1093/gastro/goae100)
Supplement: goae100_Supplementary_Data [file goae100_supplementary_data.zip › Supplemental material.docx]

**Supplemental figure 1.** Computed tomography of the abdomen. (A and B) Abdominal CT before surgery. (C and D) Abdominal CT at six months after surgery. CT, computed tomography.
