# Supplementary figures and images for: Exclusive enteral nutrition combined with continuous succus entericus reinfusion for high-output stoma in patients with Crohn’s disease: a case report
Source: Gastroenterol Rep (Oxf). 2024 Oct 27;12:goae100. doi: 10.1093/gastro/goae100 (PMC11513195; doi:10.1093/gastro/goae100)

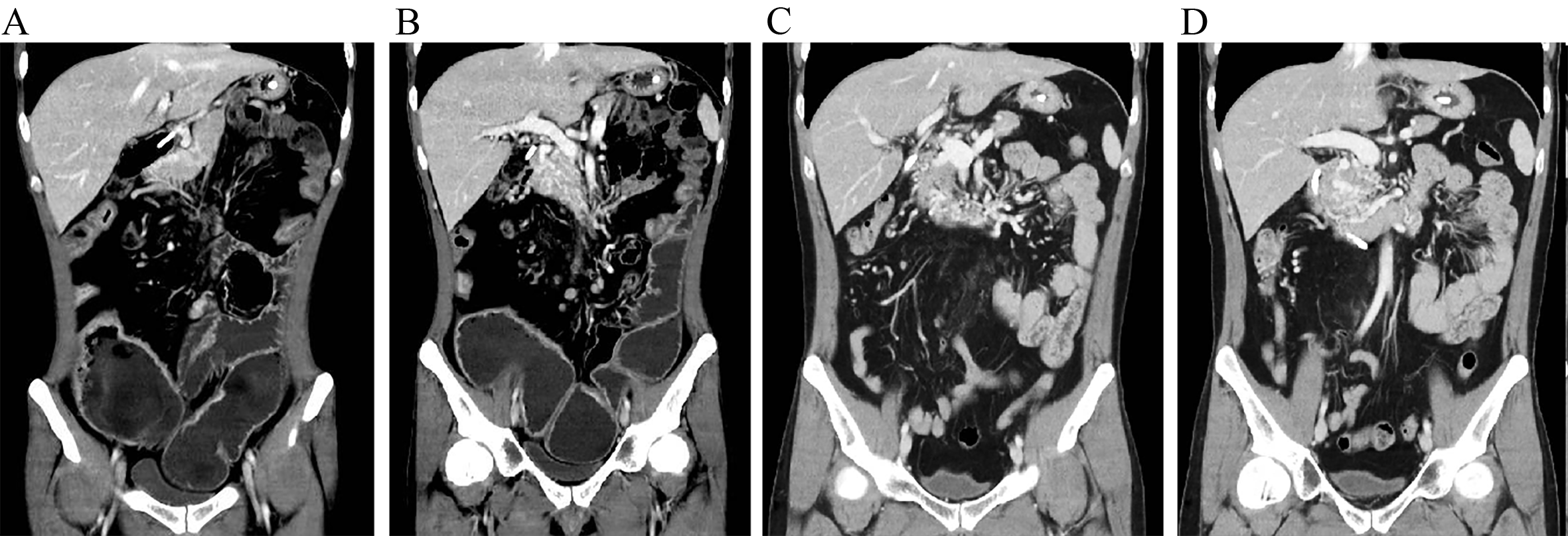

Supplement: goae100_Supplementary_Data [file goae100_supplementary_data.zip › Sup Fig 1 final version.tif]
